# A Method for Gene-Based Pathway Analysis Using Genomewide Association Study Summary Statistics Reveals Nine New Type 1 Diabetes Associations

**DOI:** 10.1002/gepi.21853

**Published:** 2014-11-04

**Authors:** Marina Evangelou, Deborah J Smyth, Mary D Fortune, Oliver S Burren, Neil M Walker, Hui Guo, Suna Onengut-Gumuscu, Wei-Min Chen, Patrick Concannon, Stephen S Rich, John A Todd, Chris Wallace

**Affiliations:** 1 JDRF/Wellcome Trust Diabetes and Inflammation Laboratory, Department of Medical Genetics, NIHR Cambridge Biomedical Research Centre, Cambridge Institute for Medical Research, University of CambridgeCambridge, CB2 0XY, UK; 2 School of Medicine, University of VirginiaCharlottesville, Virginia, United States of America; 3 Medical Research Council Biostatistics Unit, Institute of Public HealthCambridge, CB2 0SR, UK

**Keywords:** pathway analysis, genomewide association data, meta-analysis

## Abstract

Pathway analysis can complement point-wise single nucleotide polymorphism (SNP) analysis in exploring genomewide association study (GWAS) data to identify specific disease-associated genes that can be candidate causal genes. We propose a straightforward methodology that can be used for conducting a gene-based pathway analysis using summary GWAS statistics in combination with widely available reference genotype data. We used this method to perform a gene-based pathway analysis of a type 1 diabetes (T1D) meta-analysis GWAS (of 7,514 cases and 9,045 controls). An important feature of the conducted analysis is the removal of the major histocompatibility complex gene region, the major genetic risk factor for T1D. Thirty-one of the 1,583 (2%) tested pathways were identified to be enriched for association with T1D at a 5% false discovery rate. We analyzed these 31 pathways and their genes to identify SNPs in or near these pathway genes that showed potentially novel association with T1D and attempted to replicate the association of 22 SNPs in additional samples. Replication *P*-values were skewed (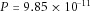) with 12 of the 22 SNPs showing 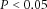. Support, including replication evidence, was obtained for nine T1D associated variants in genes *ITGB7* (rs11170466, 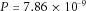), *NRP1* (rs722988, 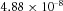), *BAD* (rs694739, 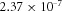), *CTSB* (rs1296023, 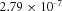), *FYN* (rs11964650, 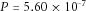), *UBE2G1* (rs9906760, 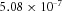), *MAP3K14* (rs17759555, 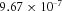), *ITGB1* (rs1557150, 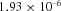), and *IL7R* (rs1445898, 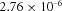). The proposed methodology can be applied to other GWAS datasets for which only summary level data are available.

## Introduction

It is increasingly recognized that pathway analysis can complement point-wise single nucleotide polymorphism (SNP) analysis in exploring genomewide association study (GWAS) data, through the identification of pathways and SNPs (genes) associated with the tested phenotype. A number of pathway analysis methods have been proposed recently that incorporate biological knowledge about genes (or SNPs) to find pathways associated with the tested phenotypes [Carbonetto and Stephens, 2013; Eleftherohorinou et al., 2009; Evangelou et al., 2012, 2013; Holmans et al., 2009; O'Dushlaine et al., 2009; Schaid et al., 2012; Wang et al., 2007, 2011; Yu et al., 2009]. These methods can be characterized by a number of aspects, including the tested null hypothesis, the input data, their test statistics and the way of assessing the significance of each pathway. One of the two null hypotheses is the competitive (enrichment) one, that states that the pathway genes are no more associated with the phenotype than the nonpathway genes. Therefore, an enriched pathway, contains more significantly associated genes than would be expected by chance. Additionally, a number of studies have been published that compared some of these methods under different settings [Evangelou et al., 2012; Tintle et al., 2009]. Evangelou et al. [2012] showed that the Fisher's method and the adaptive rank truncated product method are the most powerful methods for testing the competitive null hypothesis, in agreement with the previous literature.

One of the crucial steps of a gene-based pathway analysis is the assignment of a gene statistic that represents the association of each gene with the tested trait. Two popular statistics are the minimum *P*-value statistic and the Fisher's method statistic [Chapman and Whittaker, 2008]. Permutation procedures are needed to adjust for gene size and linkage disequilibrium (LD) between the SNPs assigned to the gene, both of which are considered to be confounding factors of pathway analysis [Evangelou et al., 2012; Wang et al., 2007]. In many cases, only summary GWAS statistics are publicly available and therefore, in this present study, we propose using genotype data available from reference panels, for example, we have used the genotype data of the WTCCC controls, for generating the null distribution of the two aforementioned gene statistics.

Several statistical models have been proposed that incorporate the pathway membership of SNPs or genes for finding SNPs associated with complex traits. Examples include the Bayesian hierarchical models proposed by Evangelou et al. [2013] and Carbonetto and Stephens [2013], and the variable selection method applied by Eleftherohorinou et al. [2009]. In the present study, we used the enriched pathways to increase our prior belief for association of SNPs near pathway genes. Instead of applying a complex statistical model for finding SNPs associated with the tested phenotype, we propose a simple procedure for prioritizing SNPs. SNPs in or near genes in enriched pathways that have small *P*-values and that have not been reported previously as associated with the tested phenotype are selected for further analyses. We propose that by using additional samples for replicating the association, novel associations can be found. We argue that SNPs with combined *P*-values less than 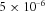 and with replication *P*-values less than 0.05 in additional cohorts are potential disease associated SNPs, because their membership of enriched pathways increases the prior belief of association.

In this study, we explored the genetic architecture of type 1 diabetes (T1D) using pathway analysis, through which pathways statistically enriched for association with T1D are defined and used to identify additional T1D loci and candidate genes. T1D is a common autoimmune disease resulting from destruction of the insulin producing beta cells in the pancreas. Genetic predisposition to T1D has been explored through linkage and association studies. The strongest genetic risk factor for T1D lies in the major histocompatibility complex (*MHC*) region (chromosome 6p21), with 49 further loci showing association (T1DBase, 05/05/2014 - Burren et al. [2011]). T1D has a classic polygenic mode of inheritance and hence many more susceptibility loci remain to be mapped, as evident, for example, from the strong linear correlation between the number of samples analyzed and the number of loci reaching genome wide significance in published studies of genetic association in autoimmune diseases [Parkes et al., 2013].

## Materials and Methods

### Materials

#### GWAS Data

The Barrett et al. [2009] meta-analysis study includes three constituent studies: WTCCC, T1DGC, and GoKinD/NIMH. The standard quality control filters applied include SNPs with minor allele frequency (MAF) greater than 0.01, less than 5% missing data, and with the Z^2^-statistic for Hardy-Weinberg equilibrium within the controls smaller than 25. In total, 822,739 SNPs were retained for analysis.

Further, the available raw genotype data of the first two GWAS were analyzed. The WTCCC GWAS was described by The Wellcome Trust Case Control Consortium [2007]. The 2,000 WTCCC cases are part of the genetic resource for investigating diabetes (GRID) collection of the JDRF/wellcome trust diabetes and inflammation laboratory (DIL) [Todd et al., 2007]. One thousand and five hundred controls of this GWAS were recruited by the WTCCC in collaboration with the UK Blood Services and the other 1,868 controls are patients with bipolar disorder included in the WTCCC study. The individuals of this study were genotyped on the Affymetrix 500K Chip. The T1DGC GWAS was first presented in Barrett et al. [2009] and includes 4,000 British cases from the JDRF/Wellcome Trust DIL collection. In addition, 4,000 controls are included from the British 1958 Birth Cohort. The individuals of this GWAS were genotyped on the Illumina 550K platform. Barrett et al. [2009] analyzed both GWAS data using imputation to combine information across the different SNP content on the different chips. The samples that passed the quality control filters applied in Barrett et al. [2009] were included in the pathway analysis presented here. In total, the WTCCC GWAS includes 1,933 cases and 3,339 controls. The T1DGC GWAS includes 3,983 cases and 3,999 controls. Similar quality control filters as the ones applied in Barrett et al. [2009] were applied to the genotype data of both GWAS, except we set a more stringent threshold for missing data of 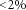.

SNPs within an extended *MHC* gene region (chr6: 25,000,000–35,000,000) were removed for all three datasets. As discussed by Elbers et al. [2009], the *MHC* region should be removed from a pathway analysis as it is a region that could potentially bias the analysis by favoring pathways related with immune functions. In T1D the causal genes in the *MHC* region have been identified as the HLA class II and class I genes and hence exclusion of the MHC region does not compromise our study.

For the purposes of this study, the genotype data of 1,350 controls recruited by the WTCCC in collaboration with the UK Blood Services, were used as the reference genotype panel for estimating the null distributions of the computed gene statistics. As discussed earlier, these controls were genotyped both on the WTCCC chip (Affymetrix 500K chip) and on the T1DGC chip (Illumina 550K platform).

#### GWAS Genes

One of the major steps of conducting a gene-based pathway analysis is the assignment of SNPs to genes. Our assignment was based on autosomal protein coding genes downloaded from Ensembl (Flicek et al. [2013], October, 2012) human assembly build GRCh37.

SNPs were mapped to genes according to their physical distance: a SNP was mapped to every gene whose coding sequence had an overlap with a 50 kb range around the SNP. In total, 18,528 overlapping genes were identified in the meta-analysis dataset. The WTCCC and T1DGC GWAS genes included were 18,353 and 18,477, respectively.

#### Pathway Databases

Three hundred and fourteen BioCarta and 1,272 Reactome [Croft et al., 2011; Matthews et al., 2009] pathways were downloaded (October, 2012). Three of the Reactome pathways did not have any of our GWAS genes. The downloaded BioCarta pathways have annotations for 1,572 genes. An average BioCarta pathway contains 17 genes and the largest pathway contains 84 genes. On the other hand, the Reactome pathways have annotations for 6,497 genes. An average Reactome pathway contains 46 genes and the largest Reactome pathway contains 1,740 genes. The two databases share 1,132 genes. Not all pathway genes are included in the lists of GWAS genes, and vice-versa. The three datasets have very similar presentation of genes for either database (Table1).

**Table 1:** Summary statistics of the database genes within the two GWAS and the meta-analysis data of Barrett et al. [2009]. The “Theoretical” represents the genes of each pathway database as these were downloaded. These numbers are reduced when SNP coverage within the studies is taken into account

Database	Study	Minimum	Median	Mean	Maximum
BioCarta	Theoretical	1	15	16.97	84
	Meta-analysis	1	13	14.79	76
	WTCCC	1	13	14.75	76
	T1DGC	1	13	14.70	76
Reactome	Theoretical	1	16.50	46.31	1,740
	Meta-analysis	0	15	37.23	1,506
	WTCCC	0	14	36.75	1,497
	T1DGC	0	15	37.15	1,496

### Methods

#### Gene Statistics

The measure that summarises the association between disease and all the SNPs assigned to a gene into a single statistic is a crucial step in a gene-based pathway analysis. A number of different gene statistics have been proposed over the years. One popular choice is the minimum *P*-value of all the SNPs assigned to the gene, i.e. the *P*-value of the most significant SNP. Chapman and Whittaker [2008] discussed that the minimum *P*-value has very good performance in cases of both low and high LD between the SNPs mapped to the gene.

An alternative, also presented in Chapman and Whittaker [2008] is the Fisher's statistic 
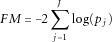
1where 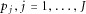 denote the single-SNP analysis *P*-values of association of the SNPs assigned to the gene with the studied phenotype. The Fisher's statistic has very good performance in cases where LD is high, but has low power in cases with no LD between the SNPs [Chapman and Whittaker, 2008]. The two tested gene statistics are denoted by (A) and (B), respectively. Gene size, i.e. the number of SNPs mapped to a gene, and the LD between the SNPs mapped to a gene can be confounding factors in a gene-based pathway analysis. In order to correct for both gene size and LD between the SNPs assigned to each gene the phenotype permutation procedure discussed in Evangelou et al. [2012] can be used.

Here, the phenotypes were permuted 1,000 times and single-SNP analysis was redone. The two aforementioned gene statistics were then recomputed for each gene. The adjusted minimum *P*-value statistic of each gene is given by 
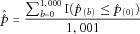
2where 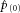 corresponds to the minimum *P*-value of the gene calculated using the observed data and 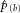 corresponds to the minimum *P*-value of the gene computed using the *b*th permuted dataset.

The corresponding adjusted statistic using the Fisher's statistic is given by 
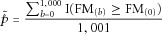
3where, similarly, 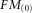 is the Fisher's method statistic calculated using the observed data and 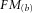 is the Fisher's method statistic computed using the *b*th permuted dataset.

Often, only summary statistics are available for published GWAS, preventing the null distribution of the two gene statistics from phenotype or SNP permutations to be computed. In this study, we propose an alternative way of computing the null distribution of the gene statistics by using the genotype data from a reference panel, motivated by the work of Liu et al. [2010] and Swanson et al. [2013] who used genotype data from the HapMap reference panels [The International HapMap Consortium, 2003] for estimating the null distribution for gene-based association tests. We, on the other hand, used the genotype data of the WTCCC controls, to secure that all GWAS genotyped SNPs are included in the study.

We repeatedly generated *Z*-statistics for all the SNPs assigned to a gene from the multivariate normal distribution with mean zero and variance Σ, where Σ is the covariance matrix of the SNP genotype data estimated from the reference genotype panel. The diagonal of Σ is set equal to 1. Subsequently, SNP *P*-values were calculated by comparing the *Z*-statistics with a Normal (0,1) distribution. Both the minimum *P*-value statistic and the Fisher's method statistic were computed for each gene. This process was repeated 10,000 times. Finally, the simulated gene statistics were compared to the original gene statistics, and adjusted *P*-values for both gene statistics were generated as described in Equations2 and 3. Here, we would like to note, that both the gene statistics were computed based on the SNPs shared between the GWAS platforms and the reference panel.

For the purposes of distinction between the statistics, the adjusted *P*-values of the gene statistics computed using the reference genotype data will be referred to as the simulated gene statistics and indicated with subscript *S*.

#### Pathway Analysis Methods

As discussed by Evangelou et al. [2012] the Fisher's method and the adaptive rank truncated product method can be adapted to test the competitive null hypothesis by using the gene statistics 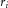. The 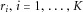 statistics equal the ranks of the genes of the study divided by the total number of genes in the study (*K*). The distribution of the gene statistics is a Uniform (0,1) and deviation from uniformity suggests enrichment of the pathway. By using the proposed gene statistics the analytic distribution of the Fisher's method and the empirical distribution of the adaptive rank truncated product method are used for testing the significance of the pathways [Evangelou et al., 2012].

**Fisher's method (FM)**. The FM statistic equals 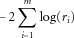
4where *m* is the pathway size. The significance of the computed FM statistic is compared with its exact χ^2^ distribution with 2*m* degrees of freedom.

**Adaptive rank truncated product method (ARTP)**. The ARTP method is a generalization of the FM where only the best *H* gene statistics within each pathway are considered for computing the rank truncated product given by 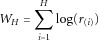
5with the gene statistics ranked from the smallest to the largest 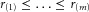. The rank truncated product combines the *H* smallest gene statistics of the tested pathway. The truncation point *H* as well as the significance of the *P*-value of the ARTP statistic were calculated using the empirical distribution of ARTP proposed by Evangelou et al. [2012].

In summary, there are eight combinations of pathway analysis methods and gene statistics (Table2). All methods were applied to the data of the T1DGC and WTCCC GWAS for comparison.

**Table 2:** The names of the methods applied to the data. FM stands for Fisher's method and ARTP stands for adaptive rank truncated product method. The gene statistics computed are the minimum *P*-value statistic and the Fisher's method statistic, which were adjusted either using a phenotype permutation procedure or using the reference genotype data for generating the corresponding SNP *P*-values

Name	Gene statistic	Procedure	Pathway analysis method
FM-(MIN)	Minimum *P*-value	Phenotype permutation	FM
FM-(FM)	Fisher's statistic	Phenotype permutation	FM
FM-(MIN_*S*_)	Minimum *P*-value	Reference genotype data	FM
FM-(FM_*S*_)	Fisher's statistic	Reference genotype data	FM
ARTP-(MIN)	Minimum *P*-value	Phenotype permutation	ARTP
ARTP-(FM)	Fisher's statistic	Phenotype permutation	ARTP
ARTP-(MIN_*S*_)	Minimum *P*-value	Reference genotype data	ARTP
ARTP(FM_*S*_)	Fisher's statistic	Reference genotype data	ARTP

#### Simulation Study

A simulation study was also performed to examine the type-I error of the methods. We aimed to compare the type-I error of the methods as well as to test how pathway size affects their type-I error. For estimating the type-I error of the methods across different pathway sizes, 1,000 random pathways of different sizes were created from the list of T1DGC GWAS genes. A 95% confidence interval for a type-I error of 5% ranges between 0.0431 and 0.0569.

#### Extending Pathway Analysis

Pathway analysis was extended for identifying genes (and SNPs) potentially associated with T1D. We searched through the *P*-values of the genes within the enriched pathways and we selected the ones that had relatively small *P*-values but have not been reported previously as associated with T1D. The SNP with the strongest association with T1D within each of the selected genes was genotyped on additional case-control and family datasets either using TaqMan or the ImmunoChip platform, a custom Illumina chip designed for dense coverage of autoimmune and autoinflammatory associated regions (Cortes and Brown [2011], ImmunoBase). We performed an inverse variance meta-analysis for combining the results of the additional cohorts. Further, using Fisher's method we combined the meta-analysis *P*-values of Barrett et al. [2009] with the *P*-values of the additional cohorts. SNPs with combined *P*-values less than 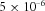 and with replication *P*-values less than 0.05 are highlighted in the Results Section. We ensured that there was no sample overlap between Barrett et al. [2009] and replication cohorts.

## Results

### Validation of Proposed Methodology

The permutation- and simulation- adjusted *P*-values were very similar for the minimum *P*-value gene statistic for both T1DGC and WTCCC GWAS, with their corresponding Spearman correlations equal to 0.9883 and 0.9690, respectively. Similarly, the Spearman correlations of the Fisher's method statistic were 0.9949 and 0.9896 for the two GWAS.

The eight methods were applied to both GWAS for comparing the agreement between the pathway *P*-values computed by each pair of competitive methods. Similarly to the observations of the gene statistics, a very high correspondence was observed between FM-(MIN) and FM-(MIN)_*S*_ for both GWAS across all 1,583 tested pathways (Spearman correlations are 0.9892 and 0.9785 for T1DGC and WTCCC, respectively). The observed correlation between FM-(FM) and FM-(FM)_*S*_ for both GWAS was similar (T1DGC ρ= 0.9823 and WTCCC ρ = 0.9735 across all 1,583 tested pathways), although lower correlations were observed between the *P*-values computed using ARTP method (Table3). Further, the simulation study showed that the type-I error of the gene statistics combined with Fisher's method is broadly maintained (Table4).

**Table 3:** Spearman correlations of the *P*-values computed for each tested pathway, for each tested database for both T1DGC and WTCCC GWAS

Database	Methods compared	Spearman correlation
T1DGC		
BioCarta	FM-(MIN) vs FM-(MIN)_*S*_	0.9939
	FM-(FM) vs FM-(FM)_*S*_	0.9755
	ARTP-(MIN) vs ARTP-(MIN)_*S*_	0.9854
	ARTP-(FM) vs ARTP-(FM)_*S*_	0.9496
Reactome	FM-(MIN) vs FM-(MIN)_*S*_	0.9878
	FM-(FM) vs FM-(FM)_*S*_	0.8378
	ARTP-(MIN) vs ARTP-(MIN)_*S*_	0.9389
	ARTP-(FM) vs ARTP-(FM)_*S*_	0.8833
WTCCC		
BioCarta	FM-(MIN) vs FM-(MIN)_*S*_	0.9761
	FM-(FM) vs FM-(FM)_*S*_	0.9739
	ARTP-(MIN) vs ARTP-(MIN)_*S*_	0.9793
	ARTP-(FM) vs ARTP-(FM)_*S*_	0.9303
Reactome	FM-(MIN) vs FM-(MIN)_*S*_	0.9784
	FM-(FM) vs FM-(FM)_*S*_	0.9729
	ARTP-(MIN) vs ARTP-(MIN)_*S*_	0.9638
	ARTP-(FM) vs ARTP-(FM)_*S*_	0.9210

**Table 4:** Type-I error of the gene statistics combined with Fisher's method for the different pathway analysis methods

	Method			
Pathway size	FM-(MIN)	FM-(FM)	FM-(MIN_*S*_)	FM-(FM_*S*_)
20	0.044	0.053	0.045	00057
50	0.043	0.041	0.043	0.044
100	0.059	0.071	0.060	0.058
200	0.053	0.054	0.048	0.049
500	0.042	0.053	0.046	0.055
1000	0.044	0.051	0.048	0.050

Given these results, we believe that the approximate null distribution is appropriate for both the FM-(MIN) and FM-(FM) methods, and we proceeded by analysing the Barrett et al. [2009] meta-analysis *P*-values using both methods.

### Results of the Meta-Analysis Study

The complete lists of enriched pathways (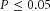) found by both methods are presented in Supplementary Tables S5–S8. We corrected for multiplicity of the tested pathways by computing the false discovery rate (FDR) *P*-values of the pathways [Benjamini and Hochberg, 1995]. Thirty-one BioCarta and Reactome pathways had FM-(MIN)_*S*_ FDR *P*-values less than 0.05, whereas 21 pathways were identified by FM-(FM)_*S*_ as enriched. As a larger number of pathways were identified by FM-(MIN)_*S*_ method, we are presenting these enriched pathways in Table5. Four of the enriched pathways of Table5, the BioCarta pathways “Adhesion molecules on Lymphocyte,” “Antigen dependent B cell activation,” “B lymphocyte cell surface molecules,” and “Lck and Fyn tyrosine kinases in initiation of TCR activation” were previously

identified by Peng et al. [2010] as enriched with T1D by analysing only the WTCCC GWAS dataset.

**Table 5:** Pathways with FDR *P*-values of FM-(MIN)_*S*_ method less than 0.05

Number	Pathway	FDR *p*-value FM-(MIN)_*S*_
i	Activation of Csk by cAMP-dependent Protein Kinase Inhibits Signaling through the T Cell Receptor	0.0436
ii	IL-2 Receptor Beta Chain in T cell Activation	0.0293
iii	HIV Induced T Cell Apoptosis	0.0106
iv	CTL mediated immune response against target cells	0.0323
v	Antigen Dependent B Cell Activation	0.0364
vi	IL-10 Anti-inflammatory Signaling Pathway	0.0115
vii	Stathmin and breast cancer resistance to antimicrotubule agents	0.0460
viii	T Helper Cell Surface Molecules	0.0021
ix	NO2-dependent IL 12 Pathway in NK cells	0.0372
x	T Cytotoxic Cell Surface Molecules	0.0014
xi	IL 17 Signaling Pathway	0.0387
xii	The Co-Stimulatory Signal During T-cell Activation	0.0003
xiii	Lck and Fyn tyrosine kinases in initiation of TCR Activation	0.0012
xiv	Role of Tob in T-cell activation	0.0414
xv	T Cell Receptor and CD3 Complex	0.0414
xvi	Selective expression of chemokine receptors during T-cell polarization	0.0395
xvii	B Lymphocyte Cell Surface Molecules	0.0375
xviii	Monocyte and its Surface Molecules	0.0460
xix	Adhesion Molecules on Lymphocyte	0.0429
xx	Double Stranded RNA Induced Gene Expression	0.0375
xxi	IFN alpha signaling pathway	0.0375
xxii	Immune System	0.0216
xxiii	Adaptive Immune System	0.0216
xxiv	Integrin cell surface interactions	0.0216
xxv	Semaphorin interactions	0.0299
xxvi	Immunoregulatory interactions between a Lymphoid and a non-Lymphoid cell	0.0012
xxvii	Effects of PIP2 hydrolysis	0.0216
xxviii	Interleukin-6 signaling	0.0445
xxix	Signal regulatory protein (SIRP) family interactions	0.0216
xxx	Catecholamine biosynthesis	0.0299
xxxi	GRB7 events in ERBB2 signaling	0.0264

### Using Enriched Pathways to Prioritize Potentially Novel T1D SNPs

We explored the gene members of the 31 enriched pathways listed in Table5 by looking at their unadjusted minimum *P*-values. We selected the genes with minimum meta-analysis SNP *P*-values less than 10^−4^ that have not been reported previously as associated with T1D (Table6).

**Table 6:** Genes of the enriched pathways that have not been reported previously as associated with T1D, but have case-control meta-analysis minimum SNP *P*-values less than 10^−4^. The most significant SNP assigned to each gene with its meta-analysis *P*-value are given in columns 3 and 4. Columns 5 and 6 present the meta-analysis *P*-value of additional cohorts if available and the combined *P*-value with Barrett et al. [2009] *P*-value, respectively. The seventh column presents any other immune (either autoimmune or autoinflammatory) disease(s) that the genes are associated with (ImmunoBase, 26/08/2014). The disease symbols correspond to: UC, Ulcerative colitis; CEL, Celiac disease; PSO, psoriasis; IBD, inflammatory bowel disease; ALO, Alopecia; and CRO, Crohn's disease. The last column presents the pathway number that each gene belongs to (Table5)

Gene	Chr	Most significant SNP	Barrett et al. [2009] meta-analysis SNP *P*-value	Additional cohorts meta-analysis SNP *P*-value	Combined *P*-value	Gene is located or is a candidate gene within an immune disease region	Pathway membership
*PSMB2*	1p34.3	rs6703605	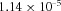				xxii, xxiii
*IL6R*	1q21.3	rs6427658	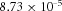	0.3260	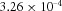	AS, JIA, RA	xxii, xxviii
*FASLG*	1q24.3	rs10912276	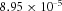	0.0290	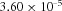	CEL, CRO, IBD	ii, iii, iv, v
*PTPRC*	1q31.3	rs2182419	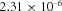	0.9593	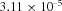	RA	i, viii, x, xiii, xvii, xxii, xxiii, xxv
*ITGA6*	2q31.1	rs16860458	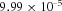				xxiv
*RAF1*	3p25.1	rs2450855	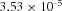	0.7642	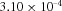		ii, xxii, xxiii
*TLR6*	4p14	rs4321646	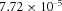				xxii
*TLR10*	4p14	rs4321646	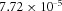				xxii
*TRPC3*	4q27	rs4502701	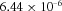	0.3697	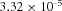		xxvii
*IL7R*	5p13.2	rs1445898	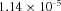	0.0146	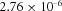	MS, PBC, UC, T1D	xxii
*DCTN4*	5q33.1	rs4246045	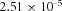	0.8456	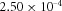	CRO, UC, IBD	xxii, xxiii
*MAPK14*	6p21.31	rs2237093	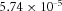				xxii
*IRF4*	6p25.3	rs2048698	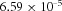	0.0296	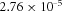	CEL, PSO, RA	xxii
*FYN*	6q21	rs11964650	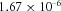	0.0183	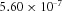	UC, CRO, IBD	xiii, xxii, xxiii, xxv
*CTSB*	8p23.1	rs1296023	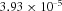	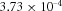	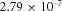		xxii, xxiii
*ITGB1*	10p11.22	rs1557150	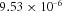	0.0119	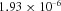		xviii, xix, xxii, xxiii, xxiv, xxv, xxvi
*NRP1*	10p11.22	rs722988	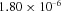	0.0013	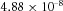		xxv
*PSMC3*	11p11.2	rs2293576	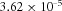	0.7537	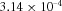	MS	xxii, xxiii
*BAD*	11q13.1	rs694739	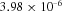	0.0031	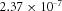	CRO, MS, UC, ALO, IBD	ii, xxii, xxiii
*AMICA1*	11q23.3	rs11216829	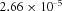	0.5807	0.0002		xxii, xxiii, xxiv, xxvi
*ITGB7*	12q13.13	rs11170466	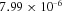	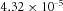	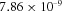		xxii, xxiii, xxiv, xxvi
*DGKA*	12q13.2	rs11171710	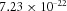				xxvii
*DNAJC3*	13q32.1	rs9302086	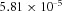				xx
*HMGB1*	13q12.3	rs1360485	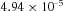	0.3819	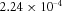		xxii
*PRKCH*	14q23.1	rs1111107	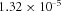			RA	xxvii
*SOCS1*	16p13.13	rs149310	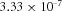	0.0064	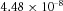	CEL, CRO, JIA, MS, PBC, PSO, UC, IBD	ii, xxii, xxiii
*TP53*	17p13.1	rs16956936	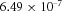	0.0834	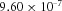		xx
*UBE2G1*	17p13.2	rs9906760	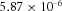	0.0047	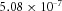		xxii, xxiii
*MAP3K14*	17q21.31	rs17759555	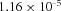	0.0047	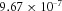	MS	xx, xxii, xxiii
*CDC34*	19p13.3	rs12982646	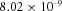	0.2323	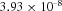		xxii, xxiii
*MADCAM1*	19p13.3	rs12982646	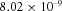	0.2323	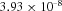		xxii, xxiii, xxiv, xxvi
*SAE1*	19q13.32	rs411560	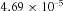			MS	xxii, xxiii

Almost 50% of our selected genes have been associated with other immune diseases. For example, *FYN* lies in regions that are associated with Crohn's disease, inflammatory bowel disease (IBD) and ulcerative colitis [Jostins and et al., 2012].*FASLG* lies in a region known to be associated with Crohn's disease and IBD [Franke et al., 2010; Jostins and et al., 2012] as well as with celiac disease [Trynka et al., 2011]. Moreover, SNP rs10912276 of *FALSG* is in perfect LD (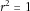) with the index SNP rs12068671 of the gene region for celiac disease (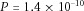, Trynka et al. [2011]).

The last column of Table6 shows the pathways that the genes belong to. The Reactome pathways “Immune system” and “Adaptive immune system” contain 24 and 17 genes of Table6, respectively, and they share 17 genes. On the other hand, 12 of the enriched pathways do not contain any of the genes presented in Table6. This suggests that the enrichment of these pathways was driven by genes already known to be associated with T1D. For example, one of them the “The Co-stimulatory signal during T-cell activation” pathway contains four genes that lie in regions associated previously with T1D: *CTLA4, IL2, ICOS*, and *PTPN11*, where *CTLA4* and *IL2* are likely causal gene candidates.

We tested whether the SNPs in Table6 were associated with T1D in additional case-control and family data. In total, genotype data for additional samples existed for 22 out of 30 selected SNPs (Supplementary Table S1). Twelve of the 22 SNPs had replication *P*-values less than 0.05 in the additional samples, an event with associated probability 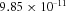, whereas just one of them would be expected to have a *P*-value less than 0.05 by chance. The skewing of the replication *P*-values toward smaller values for the SNPs of Table6 suggests that more of these SNPs are expected to replicate with larger cohorts. We identified ten SNPs with combined *P*-values less than 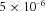 and with replication *P*-values less than 0.05 in the additional cohorts in or near the genes *IL7R, FYN, CTSB, ITGB1, NRP1, BAD, ITGB7, SOCS1, UBE2G1*, and *MAP3K14*. Although not all of these SNPs have reached genomewide significance (*P* 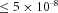), their membership of enriched pathways increases the prior for association.

SNP rs11170466 of *ITGB7* reached genomewide significance (combined *P*-value=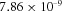, Table6) and the signal is independent of the neighboring T1D region 12q13.3 (Supplementary Table S2). SNP rs11170466 is also a *cis*-eQTL in blood cells for *ITGB7* (unadjusted *P*-value 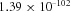), where the minor allele is associated both with increased risk of T1D and also with increased gene expression (Supplementary Table S1, [Westra et al., 2012]). The minor allele of SNP rs722988 of *NRP1* reached genomewide significance with combined *P*-value = 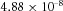.

SNP rs193779 near *SOCS1* showed a strong association with T1D (combined *P*-value= 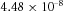). However, *SOCS1* is located very close to an established T1D locus, with SNPs in intron 19 of *CLEC16A* reported to alter T1D risk through their effect on expression of *DEXI* [Davison et al., 2011]. We conditioned on the most associated SNP in the *CLEC16A* gene region and found the association with rs193779 was considerably attenuated (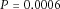; Supplementary Table S4).

SNP rs1296023 of *CTSB* showed evidence of association with T1D (combined *P*-value=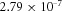; Table6). This is a novel association with T1D and the first association of T1D on chromosome 8 (T1DBase). SNP rs694739 of *BAD* also showed a strong association with T1D (combined *P*-value = 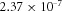). *BAD* is an interesting causal candidate gene as it overlaps with a region on chromosome 11q13.1 known to be associated with multiple sclerosis, ulcerative colitis, IBD, alopecia, and Crohn's disease (ImmunoBase). Our tested SNP rs694739 has shown convincing evidence of association with multiple sclerosis, Crohn's disease and alopecia and it is considered to be the index SNP for these three diseases in this gene region (ImmunoBase).

SNPs rs9906760, rs11964650, and rs17759555 of *UBE2G1, FYN*, and *MAP3K14* showed association with T1D with combined *P*-values less than 10^−6^. *FYN* has also been highlighted by Carbonetto and Stephens [2013] as a novel candidate T1D gene. *FYN* lies in a region of chromosome 6q21 known to be associated with Crohn's disease and ulcerative colitis [Jostins and et al., 2012]. SNP rs11964650 is not in LD with the index Crohn's disease and ulcerative colitis SNP of the region (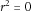 with rs3851228, ImmunoBase). The *UBE2G1* SNP rs9906760 is related with decreased expression of *cis*-eQTL in blood cells with an unadjusted *P*-value 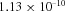 [Westra et al., 2012].

SNP rs1557150 near *ITGB1* also showed association with T1D, with combined *P*-value 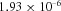 (Table6). We confirmed that the signal of SNP rs1557150 near *ITGB1* is independent of the signal of SNP rs722988 of *NRP1* (Supplementary Table S3). SNP rs1445898 of *IL7R* with combined *P*-value = 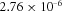 is a novel T1D association on chromosome 5.

*FYN, CTSB, BAD, ITGB7, UBE2G1*, and *MAP3K14* are members of the Reactome “Immune system” and “Adaptive immune system” pathways and are six of the 17 genes shared between the two pathways.

One SNP highlighted by our analysis is rs12982646 in *CDC34/MADCAM1*. Both genes are members of the reactome pathways “Immune system” and “Adaptive immune system,” and *MADCAM1* member of the enriched reactome pathways “Integrin cell surface interaction” and “Immunoregulatory interactions between a lymphoid and a nonlymphoid cell” (with combined *P*-value = 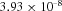). Eleftherohorinou et al. [2009] also identified *MADCAM1* as associated with T1D. rs12982646 exceeded the genomewide significance threshold in Barrett et al. [2009] but at the time were unable to test for replication of the SNP because reliable TaqMan data were not available. The replication genotype data used here, both from ImmunoChip and a new and robust TaqMan assay, did not show any evidence of association (Table6). All our samples were subsequently genotyped on TaqMan, which confirmed the fidelity of the genotype calls in the Barrett et al. [2009] study and ImmunoChip (with 99.8% genotype agreement across 6,258 samples), but also showed no overall replication in the independent samples (combined *P*-value = 0.2323). Therefore, we conclude, that this SNP is either not associated with T1D, or associated with too small an effect to be detected in our available replication samples.

## Discussion

Our results illustrate the additional biological understanding and novel genetic associations that can be revealed in existing GWAS data by pathway analysis. We have shown by comparative analysis of real datasets that our proposed methodology for obtaining the null distribution of gene statistics using reference genotype panels and summary GWAS statistics is comparable to that found by phenotype permutations when full GWAS data are available. Although motivated by published approaches to gene-based association testing, this idea has not, to our knowledge, been applied to pathway analysis. Given the difficulties that can arise accessing individual level genetic data, our method will allow broader application of pathway analyses to published GWAS. Instead of using the genotype data from the available controls, a potential alternative is the use of the freely available genotype data either from the 1,000 Genomes project [The 1000 Genomes Project Consortium, 2012] or from the HapMap project. A potential drawback of using such genotype data is the loss of some of the SNPs genotyped on the GWAS platform but not included in the reference panel.

Over the last few years, a number of pathway analyses of the WTCCC T1D GWAS data have been published [Carbonetto and Stephens, 2013; Eleftherohorinou et al., 2009; Peng et al., 2010; Wang et al., 2011]. Most of these studies reported pathways related with “Antigen processing and presentation,” “Jak-STAT signaling,” “MAPK signaling” and “Type 1 diabetes mellitus” as enriched with T1D. A number of our enriched pathways can be regarded as novel because none of the previous published pathway analyses of T1D identified them, as for example the BioCarta pathway “The Co-stimulatory signal during T-cell activation” and the Reactome pathway “Immunoregulatory interactions between a lymphoid and a nonlymphoid cell.” The differences between our results and the results of previous analyses can be characterized by the greater sample size we used and by the exclusion of the *MHC* region. Pathways such as “Antigen processing and presentation” are characterized by the inclusion of the *MHC* region. By taking into account the enrichment of *MHC* in their proposed statistical method, Carbonetto and Stephens [2013] reported the “IL-2 signaling pathway” [Geer et al., 2010; Schaefer et al., 2009] as enriched for T1D.

Carbonetto and Stephens [2013] used their model-based approach for prioritizing variants within the enriched “IL-2 signaling pathway,” their analysis of the WTCCC T1D GWAS showed seven regions of the genome to have strong evidence for association within the pathway. Three of these (*RAF1, MAPK14, FYN*) had not been reported as associated with T1D previously and were suggested by Carbonetto and Stephens [2013] as potential candidate causal genes for T1D. The three genes were also highlighted by our approach as all of them are members of the enriched Reactome pathway “Immune system.” *FYN* is a key molecule in T cells and consequently a key signaling functional candidate in the T cell mediated autoimmune process of T1D.

We extended this approach by searching SNPs assigned to genes within all enriched pathways and selected the genes with relatively small *P*-values that have not been associated with T1D previously. Equally importantly, we also genotyped the selected SNPs in additional case-control and families for finding potential new T1D associations. Through the analyses of the additional datasets we identified nine novel T1D associated genes and variants, SNP rs1111107 of *ITGB7*, SNP rs722988 of *NRP1*, SNP rs694739 of *BAD*, SNP rs1296023 of *CTSB*, SNP rs11964650 of *FYN*, SNP rs9906760 of *UBE2G1*, SNP rs17759555 of *MAP3K14*, SNP rs1557150 of *ITGB1*, and SNP rs1445898 of *IL7R*.

Both *ITGB7* and *ITGB1* encode proteins that function directly with each other in receptor–ligand interactions in the homing of T cells from blood to tissues such as the intestine and pancreas. Although we cannot be confident of the *MADCAM1* T1D association, *MADCAM1* encodes the pancreas expressed receptor for the 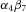 homing receptor on CD4^+^ T cells, encoded by genes *ITGA4* and *ITGB7*, and hence is a highly plausible biological candidate. Interestingly, there is a peak of SNP association, with *P*-value 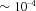, ∼300 kb 5′ of *ITGA4* in the ImmunoChip results (T1DBase, Supplementary Table S1), and the ITGB1 protein competes with ITGB7 in the 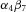 receptor [DeNucci et al., 2010]. Monoclonal antibodies against *MADCAM1* and 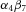 are showing clinical benefits in inflammatory bowel disease [Sheridan, 2014], and hence based on our genetic results presented here, investigation of the effects of these drugs in T1D is worth considering.

Furthermore, *CTSB*, encoding the lysosomal protease, cathepsin B, is included in the broadly defined Reactome pathways “Immune system” and “Adaptive immune system” and could participate in many processes such as apoptosis, autophagy and the NALP3 inflammsome. Nevertheless, it is not an obvious candidate gene. rs1296023 associates with *CTSB* expression in monocytes (with 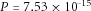, Zeller et al. [2010]), with the T1D risk allele associating with increased expression. This adds support to the possibility that *CTSB* is a T1D causal gene. This SNP or region has not been associated with any other disease (http://www.genome.gov/) or with any immune disease (ImmunoBase), which makes it interesting in that it could be unique to T1D.

*BAD* is an obvious candidate gene, encoding a key proapoptopic protein, BCL2-associated agonist of cell death, associated previously with Crohn's disease, ulcerative colitis, IBD, alopecia and multiple sclerosis, and, for example, recently shown to function in TNF-a induced apoptosis [Yan et al., 2013]. The *BAD* SNP rs694739 is the same SNP as reported for Crohn's disease, multiple sclerosis and alopecia, but only 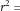
0.16 with the reported SNP for ulcerative colitis and IBD (ImmunoBase). This gene has also been associated with platelet count, via rs477895 [Qayyum et al., 2012], with very little LD between this and the T1D SNP (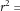 0.1, ImmunoBase). It appears that there may be multiple causal variants affecting *BAD* expression and/or function across a range of cell types. *IL7R* and *NRP1* are obvious functional candidate genes, being key molecules in the adaptive immune response [Delgoffe et al., 2013; Jäger et al., 2013]. Note that the *IL7R* signal that we report, which peaks at rs1445898 is distinct from the exonic multiple sclerosis associated SNP rs6897932 which alters splicing (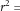
0.42) [Gregory et al., 2007].

The skewing of the replication *P*-values toward smaller values for the SNPs of Table6 suggests that more of these SNPs are expected to replicate with larger cohorts, and emphasize the potential utility of applying our proposed pathway analysis method to GWAS for which only summary statistics are available. This is also supported by the fact that some of the genes that just miss reaching our statistical threshold do show intriguing and probably meaningful links to the mechanisms of T1D. A combination of increased sample size in T1D and further GWAS coupled to the pathway analysis described here will further increase our understanding of disease mechanisms, allowing the targeting of specific genes, molecules, and cells for functional studies. The greater the number of genes and pathways accurately identified, the greater the chance of selecting pathways that might be amenable to specific therapeutic modulation. The identification of the 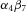 T cell adhesion pathway in T1D genetic etiology does provide a new target for potential therapeutic intervention in T1D.

## Web Resources

1. Reactome pathways: http://www.reactome.org/cgi-bin/mart
2. BioCarta pathways: http://cgap.nci.nih.gov/pathways/BioCarta_pathways
3. Ensembl: http://www.ensembl.org/index.html
4. T1DBase: http://www.t1dbase.org
5. ImmunoBase: http://www.immunobase.org
6. R package for pathway analysis: PAGWAS http://cran.r-project.org/web/packages/PAGWAS/index.html
7. Blood eQTL browser http://genenetwork.nl/bloodeqtlbrowser/

## Author Contributions

Conceived and designed the experiments: ME CW. Performed the experiments: ME. Analyzed the data: ME MDF HG JAT. TaqMan genotyping: DJS. ImmunoChip genotyping: SOG WMC PC SSR. Prepared data: OSB NMW. Wrote the manuscript: ME JAT CW. All authors have read and approved the manuscript.
